# C-Reactive Protein: An In-Depth Look into Structure, Function, and Regulation

**DOI:** 10.1155/2014/653045

**Published:** 2014-12-15

**Authors:** Juan Salazar, María Sofía Martínez, Mervin Chávez-Castillo, Victoria Núñez, Roberto Añez, Yaquelin Torres, Alexandra Toledo, Maricarmen Chacín, Carlos Silva, Enrique Pacheco, Joselyn Rojas, Valmore Bermúdez

**Affiliations:** ^1^Endocrine and Metabolic Diseases Research Center, School of Medicine, Zulia University, 20th Avenue, Maracaibo 4004, Venezuela; ^2^Institute of Clinical Immunology, University of Los Andes, Mérida 5101, Mérida, Venezuela

## Abstract

Cardiovascular disease is the leading cause of morbidity and mortality in the adult population worldwide, with atherosclerosis being its key pathophysiologic component. Atherosclerosis possesses a fundamental chronic inflammatory aspect, and the involvement of numerous inflammatory molecules has been studied in this scenario, particularly C-reactive protein (CRP). CRP is a plasma protein with strong phylogenetic conservation and high resistance to proteolysis, predominantly synthesized in the liver in response to proinflammatory cytokines, especially IL-6, IL-1*β*, and TNF. CRP may intervene in atherosclerosis by directly activating the complement system and inducing apoptosis, vascular cell activation, monocyte recruitment, lipid accumulation, and thrombosis, among other actions. Moreover, CRP can dissociate in peripheral tissue—including atheromatous plaques—from its native pentameric form into a monomeric form, which may also be synthesized *de novo* in extrahepatic sites. Each form exhibits distinct affinities for ligands and receptors, and exerts different effects in the progression of atherosclerosis. In view of epidemiologic evidence associating high CRP levels with cardiovascular risk—reflecting the biologic impact it bears on atherosclerosis—measurement of serum levels of high-sensitivity CRP has been proposed as a tool for assessment of cardiovascular risk.

## 1. Introduction

The World Health Organization currently recognizes cardiovascular disease (CVD) as the top cause of morbidity and mortality in the adult population worldwide, responsible for approximately 17 million deaths in 2008, representing 48% of global mortality from noncommunicable diseases, and with an estimated projection of 23.6 million yearly deceases by 2030 [1]. Venezuela depicts an aggravating scenario in this respect, as heart disease currently accounts for 21.36% of total national mortality [2], propelling our country to one of the highest adjusted rates of cardiovascular mortality in Latin America, with 104.2 deaths per 100,000 inhabitants during the 2003–2005 period [3].

Given the epidemic status CVD has reached worldwide and the profound impact it generates on public health systems at all levels [4], prevention strategies have become a first-line topic of scientific interest, particularly concerning risk factors and their involvement in the onset and development of disease. The Third Report of the Expert Panel on Detection, Evaluation and Treatment of High Blood Cholesterol in Adults (ATP III) [5] categorizes cardiovascular risk factors as (a) nonmodifiable, such as age, gender, and ethnicity; (b) modifiable, including diabetes mellitus, hypertension, dyslipidemia, and smoking; and (c) emerging risk factors, encompassing triacylglycerides [6], homocysteine [7], and various inflammatory markers. CRP—an acute-phase reactant—remains the most studied molecule from the latter category, exhibiting many properties which may intervene in atherogenesis [8]. Nonetheless, ongoing intense debate remains regarding its relative importance among other risk factors and its true impact on this process [9, 10]. Indeed, evidence has suggested that CRP may only potentiate vulnerability of the atheromatous plaque and formation of thrombi, rather than participate in the buildup of atheromas* per se* [11], highlighting the need for further research on CRP. This review presents the molecular basis and currently known mechanisms for the involvement of CRP in development and progression of atherosclerosis.

## 2. Overview of C-Reactive Protein Structure and Metabolism

**Figure 1:**
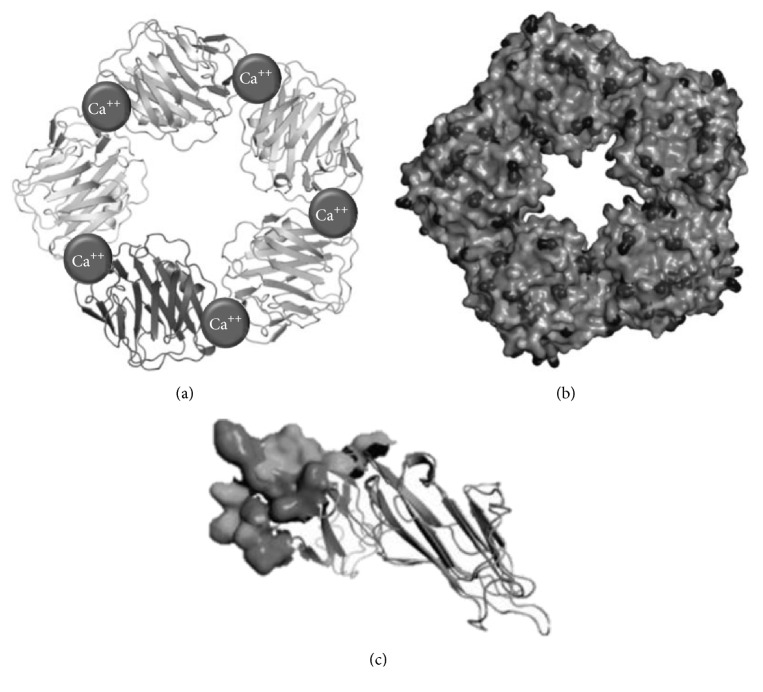
Molecular structure of C-reactive protein (CRP). (a) Tape diagram of CRP, in which the 2 Ca^2+^ atoms are presented (spheres). These are necessary for ligand binding. (b) Space model of CRP, with a phosphocholine molecule in the ligand binding site. (c) Nonglycosylated polypeptide subunit of monomeric C-reactive protein. http://www.uniprot.org/uniprot/P02741.

CRP was first described in 1930 by Tillet and Francis, named after its ability to precipitate and interact with phosphorylcholine residues of the C polysaccharide derived from teichoic acid within the cellular wall of* Streptococcus pneumoniae*, as well as its ability to precipitate with calcium ions [12]. Although CRP is classically considered an important regulator of the innate immune system and a paramount mediator of the acute-phase response [12], it has also been associated with various chronic inflammatory processes, such as certain rheumatologic conditions, cancer, and CVD [13]. CRP is a 206-amino acid member of the short pentraxin family, alongside serum amyloid P component (SAP), with high phylogenetic conservation [14]. Pentraxins share a characteristic structure: five identical nonglycosylated globular subunits—each of which is constituted by two *β*-pleated sheets—which are noncovalently associated and arranged in a symmetric cyclic pattern around a central pore, determining a pentameric, discoidal, and flattened configuration (Figure 1) [15].

C-reactive protein is predominantly synthesized in the liver (1q23.2) [16], typically within the transcriptional phase of the response to proinflammatory cytokines. IL-6 appears to be the main regulator, by promoting* de novo* synthesis of CRP via upregulation of C/EBP*β* and C/EBP*δ*, key transcription factors in this process [17]. In addition, IL-6 signaling may be reinforced by IL-1*β* and TNF, both of which increase transcription rate of* CRP* [18]. Serum CRP levels have also been closely linked to signaling by proinflammatory cytokines released by visceral adipose tissue [19]. Indeed, both hypoadiponectinemia and hyperleptinemia, two adipokine disturbances common in subjects with obesity and insulin resistance, have been linked to increased hepatic production of CRP [20, 21], as well as augmented* in situ* synthesis of CRP in vascular endothelial cells in hyperleptinemia [22].

In this regard, adipose tissue has been well characterized over recent decades as an endocrine organ, with important immunomodulatory roles through release of inflammatory messengers such as IL-1*β*, IL-6, and resistin, among others [23], underlying the chronic inflammatory component of obesity [24]. Adipocyte dysfunction is the central phenomenon in this scenario [25], encompassing hyperplasia and hypertrophy of these cells due to lipid storage increase, which leads to hypoxia, rupture propensity, and ultimately proinflammatory adipokine secretion in this tissue [25, 26]. Moreover, CRP has been shown to be expressed in adipocytes in response to proinflammatory mediators, representing yet another link between obesity and chronic inflammation [27].

Following synthesis and release into circulation, serum CRP levels tend to increase significantly 6–8 hours after initial stimulation, peaking at 24–48 hours, with a half-life of approximately 19 hours. CRP concentration in circulation is primarily determined by its synthesis rate [28]. Although the liver is the main site for production and release of CRP, its mRNA has been found in a myriad of extrahepatic sites, including adipose tissue [29], lungs [30], epithelial cells of renal cortical tubules [31], lymphocytes, and atherosclerotic lesions, in both macrophages and smooth muscle cells [32–34]. Numerous studies have focused on identifying other extrahepatic sources of CRP production that may underlie the lower and more sustained CRP concentrations which appear to predict cardiovascular risk; these include findings of CRP synthesis in coronary smooth muscle cells in response to inflammatory cytokines. Locally produced CRP may play an important role on endothelial cell activation [34].

## 3. C-Reactive Protein: From Pentameric to Monomeric

CRP has been described to adopt two different conformational forms: the native pentameric isoform (pCRP) and the monomeric isoform (mCRP) [35], which possess distinct antigenic, electrophoretic, and biological features (Figure 1) [36]. Although pCRP is the main form detected in serum [37] and appears to be a very stable molecule, current evidence suggests that conformational subunits from pCRP can be dissociated, both* in vitro* and* in vivo*, into individual mCRP units. On the other hand, independent mCRP synthesis may also be an important source of this form [38]. The two main mechanisms for* in vivo* generation of mCRP may be summarized as follows.

- Local expression: numerous studies report the presence of mCRP mRNA in various extrahepatic tissues, including adipocytes, smooth muscle cells, and inflammatory cells within atheromatous plaques. However, mechanisms for synthesis of subunits and their assembly into pCRP remain unclear. Recent* in vitro* studies support local expression, with the detection of mCRP mRNA in U937 macrophages of atherosclerotic lesions [39, 40]. In addition, greater mCRP expression has been ascertained in atherosclerotic lesions of diabetics, in association with greater systemic inflammation [41].
- Local dissociation: dissociation of pCRP into mCRP has been observed in membranes of apoptotic cells [42] and activated platelets in atherosclerotic plaques [43], representing an important interface between innate and adaptive immunity, thrombosis, and atherogenesis. Phosphatidylcholine molecules in the cell membrane of activated platelets appear to be important in this process, as this phospholipid is able to bind to circulating pCRP and induce its dissociation [44]. In a primary step of this process, a hybrid intermediate molecule termed mCRP_m_ is formed, exhibiting CRP subunit antigenicity, yet retaining native pentameric conformation. mCRP_m_ is associated with enhanced complement fixation. This molecule rapidly detaches from cell membrane and finally dissociates in solution into mCRP_s_, the final and most important form of mCRP. This second stage is associated with more powerful atherogenic properties [45].

Monomeric CRP is scarcely found in circulation through common quantification methods, suggesting a predominance of local expression, such as that seen in normal vascular tissue [46]. Nonetheless, novel techniques, such as employment of monoclonal antibodies and RNA aptamers for mCRP, allow for determination of nanomolar concentrations of this form [47–50], representing an important tool for further understanding the impact of mCRP in future research.

## 4. C-Reactive Protein: Ligands and Receptors

To date, phosphatidylcholine remains the most important CRP ligand, a phospholipid expressed in bacterial, fungal, and eukaryotic cells, particularly if these are necrotic or apoptotic, as well as in native and modified lipoproteins [51]. The CRP-binding sites for phosphatidylcholine are located on the lateral surfaces of each subunit and require binding of two calcium ions at specific hydrophobic pockets centered on Phe66, in an event known as calcium-dependent ligand binding. Then, CRP is able to interact with C1q, activating the classical pathway of the complement system [49]. CRP also has the ability to interact with other autologous ligands, such as modified and unmodified plasma lipoproteins, histones, chromatin, and small ribonucleoproteins, as well as extrinsic ligands, mainly somatic components of bacterial, fungal, and parasitic cell walls and membranes [52, 53]. Furthermore, exposure to acidic environments, such as those found at inflammation sites, triggers conformational modifications in CRP, revealing two additional binding sites within the intersubunit regions of CRP, in the loop containing residues 115–123, although the specific amino acids implicated remain unelucidated. While the first site binds factor H, the second site may bind any structurally altered protein irrespective of its identity [54].

Alongside phosphatidylcholine, pCRP may also bind to phosphocholine [55] and human complement factor H-related protein 4 (CFHR4) [56] until its dissociation into mCRP. Release of the individual subunits permits exposure of previously hidden epitopes, which entail distinct antigenic features and are capable of activating platelets, polymorphonuclear leukocytes, monocytes, lipoproteins, and the complement system* in vitro* [57].

Regarding its receptors, this protein can be recognized by immunoglobulin G (IgG) and Fc*γ*R receptors, which are cell surface glycoproteins expressed in numerous cells, including macrophages, mast cells, platelets, and leukocytes, of both lymphoid lineage and myeloid lineage [58]. Depending on the affinity with which immunoglobulins bind to the receptor, the Fc*γ*R receptors are classified either as high-affinity—known as Fc*γ*RI (CD64)—or as low-affinity receptors, such as Fc*γ*RII (CD32) and Fc*γ*RIII (CD16). These may also be further classified by their encoding genes into three distinct subgroups: a, b, and c. The signal transduction mechanism triggered by these receptors depends on activation motifs (ITAM) or inhibition motifs (ITIM) located in tyrosine-based immunoreceptors, present in cytoplasmic portions or accessory chains. The Fc*γ*RIIB is the only one of these receptors that contains an ITIM domain in its cytoplasmic portion, which allows it to negatively modulate the signaling cascade [59]. pCRP can bind specifically to Fc*γ*RI in macrophages and to Fc*γ*RIIa, in macrophages and platelets. On the other hand, mCRP isoform can only interact with the Fc*γ*RIII receptor, expressed primarily in platelets and endothelial cells [60] (Table 1).

Recent reports have commented on the CRP-binding capacity of lectin-like oxidized low density lipoprotein receptor-1 (LOX-1), confirmed through several methods, including immunofluorescence [61, 62]. This phenomenon has been linked to induction of complement activation, leukocyte infiltration, and modification of vascular response to vasodilators [63, 64], representing a shared pathway for CRP and oxidized LDL (LDL-ox) in endothelial dysfunction [65]. CRP has also been described to induce secretion of a soluble isoform of LOX-1 (sLOX-1) in classically activated macrophages and in macrophages derived from peripheral blood mononuclear cells of patients with acute coronary syndrome, in a process which appears to involve Fc*γ*RIIa, TNF, and ROS production [66]. Moreover, similar observations have been made in current smokers [67] and patients with stable coronary disease [68]. These observations outline possible clinical applications for LOX-1, as a prognostic factor in acute coronary syndrome [69] and as a potential pharmacological target, with a report describing reduced vascular toxicity induced by CRP and LOX-1 following treatment with atorvastatin [70].

## 5. The Role of C-Reactive Protein in the Pathophysiology of Atherosclerosis

**Figure 2:**
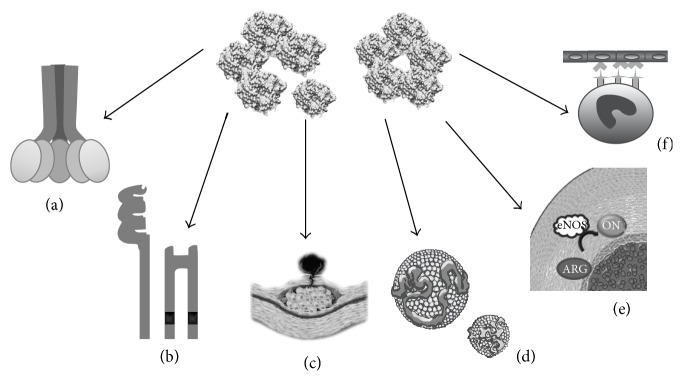
Key participations of C-reactive protein in atherosclerosis. (a) Activation of the complement system. (b) Activation of different receptors in inflammatory cells. (c) Extracellular matrix remodeling. (d) Interaction with lipoprotein. (e) Impaired nitric oxide synthesis. (f) Cell recruitment (see text for further details).

Inflammatory mechanisms play a fundamental role in all phases of atherosclerosis, from the initial recruitment of circulating leukocytes to the rupture of unstable plaques [71]. Among multiple inflammatory biomarkers, CRP boasts the largest body of research supporting its role as an independent risk factor in the development of CVD [15–19], as it actively participates in atherogenesis by directly influencing processes such as activation of the complement system, apoptosis, vascular cell activation, monocyte recruitment, lipid accumulation, and thrombosis. Both isoforms are involved in such processes: pCRP can generate inflammatory responses binding to the phosphatidylcholine on the exterior of LDL-ox and the surface of apoptotic cells [53], while mCRP is able to modulate platelet function inducing aggregation and contributes to atherothrombotic complications by promoting thrombosis [60] (Figure 2).

### 5.1. Activation of Complement System

The complement system is a set of enzymes and bioregulators with multiple biological activities, playing a key role in both innate (alternative and lectin pathway) and acquired (classical pathway) immunity. Its proper activation is essential for defense against pathogens and elimination of apoptotic and necrotic cells. Nevertheless, excessive or inappropriate activation of this system contributes to the pathogenesis of many chronic inflammatory diseases, including atherosclerosis [72]. Both CRP isoforms have the ability to interact with C1q, activating the classical pathway [73, 74]. Recent studies report the presence of CRP mRNA and depots with high concentrations of C1q, C3, and C4 in atherosclerotic plaques [75], suggesting that CRP may amplify and facilitate activation of the classical pathway, with the subsequent formation of the membrane attack complex (MAC). Furthermore, activation of this pathway may contribute to the establishment and progression of atherosclerosis by inducing the proliferation of arterial smooth muscle cells and increased synthesis and secretion of IL-8 and monocyte chemotactic protein (MCP-1). CRP also participates in the activation of nuclear factor kappa B (NF-*κ*B), a transcription factor present in rapid-response cells involved in immune and inflammatory reactions, increasing the production of cytokines, chemokines, adhesion molecules, growth factors, and immunoreceptors in several cell types within the atherosclerotic plaque [76]. CRP also exhibits affinity with members of the factor H protein family, providing binding sites for factor H in the mCRP isoform, to which it binds in a calcium-independent fashion [77]. Conversely, CFHR4 can bind to pCRP through a calcium-dependent mechanism [56]. These proteins may facilitate recruitment of CRP to the surface of necrotic cells, acting as soluble regulators of the alternative pathway of the complement system [78]. Excessive activity of this pathway leads to the presence of high circulating levels of C3a and C5a, potent anaphylatoxins involved in local inflammatory responses [79].* In vitro* studies have shown expression of CR3 and CR5a in coronary artery atherosclerotic plaques, favoring chemotaxis of monocytes, mast cells, and lymphocytes, expression of endothelial adhesion molecules, release of TNF and IL-1, and production of reactive oxygen species by macrophages located within the lesion [80, 81].

### 5.2. Interaction with Cellular Receptors

The Fc*γ*R family includes major CRP targets, expressed in numerous cells of the immune system, controlling activation, proliferation, phagocytosis, degranulation, and cytokine secretion [82], thus regulating local inflammatory processes, such as those occurring in atherosclerotic plaques. Both CRP isoforms can bind to Fc*γ*RI, Fc*γ*RIIA, and Fc*γ*RIII, inducing conformational changes in their structures that trigger intracellular signaling cascades through their ITAM motifs. Generally, this cascade would start with the sequential activation of a tyrosine protein kinase (PKT) of the Src family, which phosphorylates tyrosine residues of this motif, followed by activation of Syk tyrosine kinase [83]. This would result in the recruitment of multiple signaling molecules, including other kinases such as protein kinase C (PKC), extracellular signal-regulating kinases (ERK), mitogen activating protein kinases (MAPK) [84], and phosphatidyl inositol-3-kinase (PI3K), as well as phospholipase C (PLC) [85], intracellular adaptation molecules, and second messengers such as calcium (Ca), diacylglycerol (DAG), and inositol-3-phosphate (PI3) [85, 86]. However, recent evidence suggests that once bound to the Fc*γ*RIIb receptor—which has ITIM inhibitory intracellular domains—in endothelial cells, CRP starts a cascade characterized by the activation of phosphatases, such as SHIP-1, which then inactivate and regulate the signaling molecules previously described [87].

### 5.3. Immune Cell Recruitment, Modulation, and Activation

Recent reports suggest that pCRP may be a direct regulator of endothelial cell activation and dysfunction, by inducing the expression of intracellular adhesion molecules, vascular E-selectin, and monocyte chemoattractant protein-1 (MCP-1) [88], which permits chemotaxis and binding of monocytes to endothelial cells during the early stages of atherogenesis [89]. Moreover, CRP may drive human monocyte differentiation towards a proinflammatory M1 phenotype, mediated by Fc*γ* receptors and the NF-*κ*B pathway, as well as inducing a shift in cytokine secretion patterns in M2 macrophages towards a M1-like proinflammatory phenotype [90]. Additionally,* in vitro* studies indicate that CRP at concentrations <10 *μ*g/mL is linked to a decrease in the synthesis of prostaglandin F-1*α*, a stable metabolite of prostacyclin which regulates important endothelial processes such as vasodilation, platelet aggregation, and smooth muscle cell proliferation [91]. Similarly, CRP may generate overexpression of angiotensin type 1 receptor II (ATR-1) through the MAPK and NF-*κ*B pathways, favoring proliferation, remodeling, and migration of smooth muscle cells in atherosclerotic lesions [88, 92]. Furthermore, CRP isoforms play an important differential role in the modulation of endothelial progenitor cell (EPC) proliferation. While pCRP appears to favor EPC proliferation and induce primarily noninflammatory gene expression of these cells, mCRP does not seem to affect EPC proliferation rate but rather induce upregulation of proinflammatory, interferon-responsive genes [93]. Ultimately, the dissociation of pCRP into mCRP may be viewed as a “master switch” for the focalization of the inflammatory processes involved in the formation of the atherosclerotic plaque: by binding to phosphorylcholine in the membrane of activated platelets, pCRP is able to localize its dissociation to the vicinity of the plaque, leading to an* in situ* accumulation of mCRP, which favors the further development of the plaque by all previously described mechanisms [94, 95].

### 5.4. Activation of Metalloproteinases

Metalloproteinases are proteolytic enzymes responsible for remodeling the extracellular matrix (ECM), which have been implicated in the development and rupture of atherosclerotic plaques.* In vitro* and* in vivo* studies have demonstrated CRP to augment expression of metalloproteinase-1 (MMP-1) and metalloproteinases 1, 2, and 9 via p38-MAPK, ERK, and JNK signaling [96–98].

### 5.5. Nitric Oxide Synthesis

Nitric oxide (NO) is a simple gas produced by a group of enzymes termed NO synthases. These are widely distributed in several tissues, particularly in endothelial cells, where they mediate vasodilation and antioxidant and antithrombotic effects [99].* In vitro* and* in vivo* studies indicate that CRP may interfere with NO synthesis by inhibiting endothelial nitric oxide synthase (eNOS) activity through various pathways, all of which ultimately lead to endothelial dysfunction [100]. To this end, CRP has been demonstrated to inhibit GTP cyclohydrolase 1 through the p38 kinase pathway [101]; this enzyme is the first step in the* de novo* synthesis of tetrahydrobiopterin, an important cofactor for eNOS. As a result, decreased tetrahydrobiopterin leads to decoupling of eNOS and depletion of NO levels, favoring endothelial dysfunction. Additionally, Fc*γ* receptors, as well as CD32 and CD64 receptors, have been shown to mediate a decrease in the phosphorylation of eNOS in Ser1177 and an increase in phosphorylation of Thr495, resulting in diminished eNOS activity [102]. Several reports indicate that inhibition of this enzyme may be mediated specifically by Fc*γ*RIIb through the phosphatase 2A pathway, preventing bradykinin- and insulin-triggered phosphorylation of eNOS [103, 104]. Yet another mechanism contributing to endothelial dysfunction involves modification of protein-protein interactions of eNOS with heat shock protein 90 (Hsp90) and caveolin-1, decreasing binding to the former and increasing binding to the latter, resulting in reduced eNOS activity [105].

### 5.6. Lipoproteins

**Figure 3:**
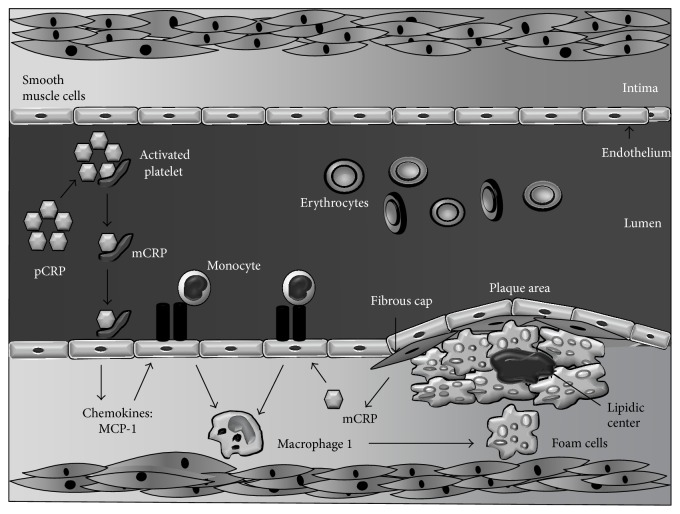
Role of C-reactive protein in the arterial intima during atherosclerosis. C-reactive protein is a cardiovascular risk factor that plays an important role in atherosclerotic events, found in unstable plaques in the vascular endothelium, along with other proatherogenic components. Binding of pCRP to activated platelets results in generation of mCRP, which can then enhance platelet adhesion to endothelial cells and stimulate formation of neutrophil-platelet aggregates, favoring thrombogenesis (see text for further details).

mCRP selectively binds to low density lipoprotein (LDL) and, in a lesser proportion, to very low density lipoproteins (VLDL) [52, 106], while pCRP interacts mainly with highly immunogenic forms of these lipoproteins, such as LDL-ox, enzymatically altered LDL (E-LDL), and minimally modified LDL (mmLDL) [107, 108]. Microenvironmental pH at the inflammation site plays a key role in the binding of CRP to lipoproteins, as the binding site for LDL-ox is only revealed after modifications in the structure of CRP triggered by acidic milieus [109, 110]. Furthermore, despite being able to bind E-LDL at physiological pH, acidic pH enhances affinity for this modified lipoprotein [51]. This association is thought to lead to opsonization and subsequent phagocytosis of LDL-ox and, in consequence, formation of a characteristic component of atherosclerotic plaques: foam cells [111] (Figure 3).* In vivo* assays have demonstrated that CRP not only promotes uptake of LDL-ox but also stimulates accumulation of cholesterol esters in human macrophages [112]. Likewise, pretreatment with anti-CD32, CD36, and CD64 diminishes CRP activity, suggesting involvement of both Fc*γ* and scavenger receptors [113]. Lastly, although pCRP may exert some anti-inflammatory effects by binding mmLDL and consequently attenuating monocyte activation, this property is lost when dissociated into mCRP, further highlighting the importance of this dissociation as a localized inflammation mechanism [114, 115].

## 6. Pentameric versus Monomeric C-Reactive Protein

Each isoform appears to play distinct roles throughout the atherosclerotic process, and both are subjects of continuous study. Nevertheless, mCRP appears to play a more direct or “effector” role in atherosclerosis, in contrast to pCRP, which may be described as a “facilitator” in circulation, awaiting dissociation for focalization of proinflammatory effects to injured sites, such as atheromatous plaques [88, 93]. In this context, the only relation between pCRP and activated platelets seems to be its dissociation into mCRP on their surface, as only the latter favors thrombosis in this scenario by promoting platelet aggregation, surface P-selectin and CD63 exposure, and glycoprotein IIb-IIIa activation [116].

Likewise, mCRP induces expression of tissue factor in endothelial cells, favoring fibrinolytic resistance and endothelial dysfunction [117], and is also much more effective than pCRP at inducing chemotaxis and binding to integrin in macrophages, as well [118]. Indeed, current knowledge depicts mCRP to exhibit more deleterious actions than pCRP and seems to be more powerful regarding the effects they share in atherosclerosis [38], although further research may reveal novel aspects in the properties of both isoforms that may modify this outlook.

## 7. Conclusions

The concept of atherosclerosis has long diverged from mere lipid deposition in arterial walls, towards the current notion that describes it as a complex chronic inflammatory process. In this scenario, CRP may play an active role through a wide array of mechanisms, although primarily via activation of the complement system and metalloproteinases, and recruitment and activation of inflammatory cells. Simultaneously, CRP favors the establishment of a generalized chronic inflammatory state and in turn potentiating atherosclerosis. Although mCRP appears to drive most of these effects, further research is required in order to differentially characterize the roles of CRP isoforms.

Moreover, many epidemiological studies have shown an association between CRP and cardiovascular risk [119], and its clinical utility is currently a topic of great debate, with novel proposals for scenarios where its evaluation may be useful, including the use of fractions of CRP as a new element in the diagnostic workup of patients with acute coronary syndrome [120] and the designation of CRP as a potential therapeutic target given its exhaustive involvement in the pathophysiology of atherosclerosis [121]. In this context, the results from the JUPITER study offer some insight into this possibility, by ascertaining better health outcomes in patients with lower CRP levels [122]. Thus, further experimental testing is required to elucidate its true involvement as a risk factor, as well as population studies exploring the epidemiologic behavior of CRP.

## Figures and Tables

**Table 1:** Receptors, ligands, and function of C-reactive protein according to location.

Receptor	Ligands	Location	Function	Author (reference)
Fc*γ*RI (CD64)	pCRP	Monocytes	Induces release of cytokines	Li et al. [61]
		Macrophages	Mediator of phagocytosis	Shih et al. [62]

Fc*γ*RIIa (CD32A)	pCRP	Monocytes	Induces release of cytokines	Li et al. [61]
		Macrophages	Induces expression of LPL Mediator of phagocytosis	Li et al. [61]; Shih et al. [62]
		Platelets	Inhibits binding of platelets to neutrophils	Fujita et al. [63]
		PMN	Inhibits expression of CD62L	Fujita et al. [63]

Fc*γ*RIIb (CD32B)	pCRP mCRP	Endothelial cells	Inhibits bradykinin- and insulin-mediated activation of eNOS	Hein et al. [64]

Fc*γ*RIII (CD16)	mCRP	Monocytes	Induces release of cytokines	Fujita et al. [63]
		Macrophages	Induces expression of LPL	Shih et al. [62]; Boncler et al. [60]
		Platelets	Promotes binding of neutrophils	Fujita et al. [63]
		Endothelial cells	Induces synthesis of IL-8 and MCP-1 Induces expression of endothelial adhesion molecules	Li et al. [61]; Fujita et al. [63]

LOX-1	pCRP	Human aortic endothelial cells (*in vitro *models)	Increases human monocyte adhesion and LDL-ox uptake to endothelial cells	Szalai [65]
